# The Treatment of Gall Stones

**Published:** 1902-05-24

**Authors:** 


### THE TREATMENT OF GALL STONES.

In all diseases in which the symptoms, severe and distressing though they be, are interrupted by periods of complete remission, the hopefulness of the patient often stands in the way of the adoption of the best form of treatment, for when once the remission is established the temptation is strong to put off all serious measures until a more convenient season. In few maladies is this difficulty more marked than in cases of gall stones. Commenting upon a series of cases of this nature, Dr. A. A. Berg,[1] of New York, asks the question, What are the indications for surgical intervention in biliary lithiasis? Before answering this question, he insists upon the important bearing which the occurrence of any degree of inflammation has upon the problem. An attack of biliary colic is due to attempts on the part of an irritable viscus to expel a foreign body, and these should give rise only to pain and the reflex symptoms depending upon irritation of the sensitive nerves. Fever is always absent; in fact, the temperature is apt to be subnormal. Pyrexia and distension of the gall bladder are symptoms of a secondary invasion of the biliary system by virulent organisms and Dr. Berg maintains that this event completely alters the whole nature of the disease and its treatment. The first attack of cholecystitis, accompanying calculous disease of the gall bladder or ducts, calls then for the abandonment of the medical treatment and a resort to surgical intervention in order to remove the calculi and cure the cholecystitis. His general conclusions then may be stated as follows:—*Indications for Medical Treatment.*—Cholecystitic pain or attacks of biliary colic, in either case unattended with fever. *Indications for Surgical Treatment.*—(1) Operations of choice, undertaken in the quiescent period with the object of avoiding serious complications; simple procedures followed by a mortality of 2 to 3 per cent., (*a*) severe cholecystitic pain or oft-repeated uncomplicated attacks of biliary colic, persisting in spite of medical treatment, and in virtue of which the patient becomes invalided and incapacitated for work; (*b*) after the first attack of acute cholecystitis associated with fever. (2) Compulsory operations, undertaken often amidst unfavourable surroundings and in patients who are septic, emaciated, and of low vitality; difficult and laborious procedures, attended with a high mortality; (*a*) intensely acute attacks of cholecystitis, which may be the first indication of calculous disease, but usually follows milder attacks; (*b*) hydrops, empyema, gangrene, or perforation of the gall bladder, cholæmia, abscess of the liver, and diffuse peritonitis.

[1] New York Medical Record, May 3.

